# Coproscopy and molecular screening for detection of intestinal protozoa

**DOI:** 10.1186/s13071-017-2346-7

**Published:** 2017-09-06

**Authors:** Marawan Abu-Madi, Sonia Boughattas, Jerzy M. Behnke, Aarti Sharma, Ahmed Ismail

**Affiliations:** 1 Department of Biomedical Science, College of Health Sciences, Biomedical Research Center, Qatar University, P.O. Box 2713, Doha, Qatar; 2 School of Life Sciences, University of Nottingham, University Park, Nottingham, NG7 2RD UK; 3 Medical Commission, Ministry of Public Health, P.O. Box 42, Doha, Qatar

**Keywords:** Intestinal protozoa, New immigrant, Food-handlers, Renewal applicant, RT-PCR, Coproscopy, Qatar

## Abstract

**Background:**

Intestinal parasitosis is one of several health concerns about immigrants who travel from endemic to non-endemic regions. Reliable rapid sensitive diagnostic tools, for use in non-endemic regions, are urgently required to enable frequent assessment of immigrant workers in jobs where risk of local transmission is a particular concern (e.g. food-handlers). We assessed the burden of intestinal protozoa in newly arrived immigrants and those applying for renewal of work permits in Qatar (*n* = 735), by both microscopic examination of stool samples and by Real Time PCR methodology.

**Results:**

Prevalence was considerably higher using RT-PCR compared with coproscopy (*Blastocystis hominis*: 65.2 *vs* 7.6%; *Giardia duodenalis*: 14.3 *vs* 2.9%; *Entamoeba histolytica*: 1.6 *vs* 1.2%). *Dientamoeba fragilis* was sought only by RT-PCR (prevalence of 25.4%). Prevalence of *G. duodenalis* was significantly higher in male subjects, associated with blue collar workers and declined over time. Prevalence of *B. hominis* varied significantly with region of origin of subjects with highest values recorded among African immigrants. Prevalence of *D. fragilis* also varied with region of origin of subjects, and was lower in young female subjects and in renewal applicants compared with first-time applicants for work permits.

**Conclusions:**

We strongly recommend that, henceforth, intestinal protozoa should be screened by RT-PCR, with a particular focus on frequent assessment of immigrant food-handlers.

## Background

The World Health Organization estimates that about 3.5 billion people worldwide are affected by intestinal parasitic infections [1], notably those living in developing countries. Enteric parasitoses may be spread by immigrant workers from endemic parts of the globe, seeking jobs in more developed parts of the world, where parasitic infections are mostly less prevalent. It is therefore important that countries that receive immigrants to have monitoring systems for these infections in place [2]. This should encompass both assessment of all asymptomatic immigrants on first arrival and regular re-assessment thereafter over appropriate periods of time, especially if home visits occur frequently [3]. The prevalence of gastrointestinal parasites is known to vary from one region to another, depending on a range of socioeconomic and intrinsic factors (e.g. the degree of personal and community hygiene, sanitation, age profile of the population, standard of living) and extrinsic factors, such as local climate and nature of the environment. Moreover, different diagnostic techniques implemented locally by health authorities may also differ in their detection efficiency and thereby affect local health statistics [4].

Diagnosis of enteric parasitic infections is achieved primarily by the traditional microscopic examination of stool samples (coproscopy). This is still regarded as the gold standard when performed by an experienced and highly skilled microscopist. However, the sensitivity and specificity of coproscopy for detection of protozoan parasites are now both regarded as less than desirable, the technique being limited by its poor sensitivity and inability to differentiate between closely related species. For example, it is impossible to differentiate microscopically between the cysts of the pathogenic and the non-pathogenic species of *Entamoeba histolytica* and *Entamoeba dispar* [5]. It is also of some concern that currently, there is a world-wide shortage of skilled technologists capable of reliably detecting these infections by coproscopy. As the baby boomer generation retires from the workforce, inexperienced technologists, who in some instances are inadequately trained in parasitology, are left to fill the void [6].

Novel, rapid and sensitive diagnostic tests, which may be used in non-endemic areas with minimal training, are urgently required. Molecular approaches based on the polymerase chain reaction (PCR) are becoming increasingly available for detecting intestinal parasites, demonstrating higher sensitivity and specificity with respect to conventional methods such as coproscopy [7]. The application of real-time PCR (RT-PCR) in molecular diagnostics has boosted the use of nucleic acid-based detection methods with accurate sensitivity and fewer manipulation steps associated with the procedure [8]. Notable differences were reported when comparing coproscopy and RT-PCR sensitivity in detecting for example *Giardia duodenalis* [9] and *Blastocystis hominis* [10]. In an Australian study focusing on *Dientamoeba fragilis*, RT-PCR detected 35 positives among 650 samples, while coproscopy detected only 12 positives [11]. Prevalence of *E. histolytica*/*dispar* was found to be 9.2% based on coproscopy *vs* 14.3% according to the results of PCR elsewhere [12].

An important aspect of risk management of intestinal parasitic infections in the community concerns the nature of some jobs [13], and in this context immigrants working in the food service settings are a particular concern. Indeed if they are infected by enteropathogens, and if appropriate personal hygiene and sanitary behavior are not adopted, then fecal contamination during food preparation can occur *via* contaminated hands. Therefore, an efficient and reliable screening procedure for food-handlers, alongside staff training in thorough hand washing and an emphasis on hygiene, should be mandatory in countries where immigrant workers are employed in the food industry in order to ensure protection of consumer health [14].

In Qatar, the most widely used methods for surveillance of enteric infections among newly arrived and resident workers still rely on coproscopy [15]. In this paper we assess a method based on PCR as an alternative to examination of stool samples by microscopy. We identify some remaining problems with this technology and we use data based on RT-PCR to assess some of the factors that influence the prevalence of intestinal protozoan infections among immigrants.

## Methods

### Samples

Stool samples were collected and processed as described in detail previously [15]. Samples were aliquoted for conventional coproscopy alongside DNA extraction using the QIAamp DNA stool minikit (Qiagen, Hilden, Germany), according to the manufacturer’s instructions. Sample analysis was done by RT-PCR as described elsewhere [5, 10] using the following primers/probe sets: For *Blastocystis hominis*, 5′-GCT CCG GTG AAG ACT TTG GAT TT-3′ as forward primer and 5′-CCT ACG GAA ACC TTG TTA CGA CTT CA-3′ as reverse primer with SyberGreen dye; for *Giardia duodenalis*, 5′-GAC GGC TCA GGA CAA CGG TT; 5′-TTG CCA GCG GTG TCC G-3′ and FAM-5′-CCC GCG GCG /ZEN/ GTC CCT GCT AG-3′; for *Entamoeba histolytica*, 5′-ATT GTC GTG GCA TCC TAA CTC A-3′; 5′-GCG GAC GGC TCA TTA TAA CA-3′ and HEX-5′-TCA TTG AAT /ZEN/ GAA TTG GCC ATT T-3′; and for *Dientamoeba fragilis*, 5′-GTT GAA TAC GTC CCT GCC CTT T-3′; 5′-TGA TCC AAT GAT TTC ACC GAG TCA-3′ and FAM-5′-CAC ACC GCC CGT CGC TCC TA-3′ as a probe.

### Inhibition test

In cases of conflicting results (samples positive by coproscopy and negative by RT-PCR), inhibition was checked. The RT-PCR experiments were re-run using controls added post-extraction but pre-amplification by spiking an aliquot of the specimen matrix with a positive DNA and then subjecting it to amplification. Moreover, RT-PCR procedure modification included tenfold dilution of the sample DNA and the addition of non acetylated Bovine Serum Albumin (BSA) (Thermo fisher Scientific, Massachusetts, USA) to the RT-PCR master mix at 400 ng/μl.

### Statistical analysis

To test for concordance between the outcome of RT-PCR and coproscopy, we used the Related-Samples McNemar’s Change Test (non-parametric test) in SPSS v. 23.

Prevalence data are shown with 95% confidence limits (CL_95_), calculated as described elsewhere [16] employing bespoke software. Prevalence was analysed by maximum likelihood techniques based on log-linear analysis of contingency tables using the software package IBM SPSS Statistics (Version 23). The analysis was based on data recorded at the Medical Commission from the 1st January 2012 to 31st December 2014. Initially, full factorial models were fitted, incorporating as factors sex (SEX at 2 levels, males and females), year (YEAR at 3 levels for each of the years: 2012 [*n* = 196]; 2013 [*n* = 208]; and 214 [*n* = 331]), age (AGE CLASS at 4 levels: Age class 1 [18–22 years, *n* = 88]; Age class 2 [23–29 years, *n* = 266]; Age class 3 [30–37 years, *n* = 227]; and Age class 4 [38–58 years, *n* = 154]), region of origin (REGION at 4 levels, as defined below) and job/profession (JOB FAMILY at 5 levels: Blue collar workers [mechanics, masons, builders, car wash attendants, carpenters, cleaners, crane operators, drivers, electricians, fire fighters, fitters, gardeners, labourers, painters, plumbers, steel fixers, welders, *n* = 273]; Pink collar workers [barbers, beauticians, butlers, grocers, hairdressers, life guards, merchandisers, nurses, safety officers/guards, sales persons, saloon workers, security guards, tailors, *n* = 46]; White collar workers [accountants, cashiers, civil engineers, clerks, IT experts, office boys, receptionists, secretaries, *n* = 20]; Food-handlers [bakers, butchers, chefs, cooks, kitchen assistants, waiters/waitresses, *n* = 43]; and housemaids [*n* = 353]). The pink and white collar worker job families were combined for the present analysis (*n* = 66) because their sample sizes were small.

The study population (*n* = 735) came from 18 countries which we allocated to four geographical regions for ease of initial analysis. These were Eastern Asia (*n* = 223) comprising Indonesia (*n* = 136), Philippines (*n* = 83), Thailand (*n* = 2) and Vietnam (*n* = 2); Western Asia (*n* = 423) comprising Bangladesh (*n* = 87), India (*n* = 141), Nepal (*n* = 97) and Sri Lanka (*n* = 98); northern and Saharan Africa (*n* = 42) comprising Chad (*n* = 1), Egypt (*n* = 2), Eritrea (*n* = 3), Ethiopia (*n* = 34) and Sudan (*n* = 2); sub-Saharan Africa (*n* = 47) comprising Cameroon (*n* = 1), Ghana (*n* = 3), Kenya (*n* = 27), Nigeria (*n* = 15) and Uganda (*n* = 1).

In some analyses we also fitted IMMIGRATION STATUS. At interview we recorded whether a person was applying for a work permit for the very first time (indicating that he/she was a freshly arrived immigrant, seeking work in Qatar for the first time and therefore not resident in the country in the preceding months) or applying for renewal of their permit (suggesting that they had already been resident in Qatar for at least the last 1 year). For the latter group we also recorded the number of times a person had applied for renewal of their work permit. This ranged from first renewal to twelfth.

For each species of parasite in turn the presence/absence of parasites (INFECTION) was coded as a binary factor. The explanatory factors listed above were fitted initially to all models that were evaluated. For each level of analysis in turn, beginning with the most complex model, involving all possible main effects and interactions, those combinations that did not contribute significantly to explaining variation in the data were eliminated in a stepwise fashion beginning with the highest-level interaction (the backward selection procedure). A minimum sufficient model was then obtained, for which the likelihood ratio of *χ* ^2^ was not significant, indicating that the model was sufficient in explaining the data. The importance of each term (i.e. interactions involving infection) in the final model was assessed by the probability that its exclusion would alter the model significantly and these values relating to interactions that included presence/absence of infection (INFECTION) are given in the text. Where relevant, we also fitted in turn models with just one of the factors and INFECTION.

## Results

### Relationship between detection of positives by coproscopy and by PCR

As assessed by RT-PCR, the number of detected positives of most species was considerably higher than when detection was based on faecal cyst counts using coproscopy (Table 1). However, some positives were detected only by coproscopy (Table 2).

**Table 1:** Prevalence ﻿% (CL_95_)﻿ of intestinal protozoa, based on detection by coproscopy and RT-PCR

Species	PCR	Coproscopy	Both methods^a^
*Entamoeba histolytica*	1.6 (0.92–2.88)	1.2 (0.63–2.36)	2.6 (1.63–4.06)
*Giardia duodenalis*	14.3 (11.85–17.09)	2.9 (1.83–4.39)	15.5 (12.98–18.39)
*Blastocystis hominis*	65.2 (61.55–68.64)	7.6 (5.84–9.84)	66.3 (62.66–69.71)
*Dientamoeba fragilis*	25.4 (22.33–28.33)	na	na

^a^Based on positives detected by both methods + positives detected only by RT-PCR + positives detected only by coproscopy

*Abbreviation*: *na* not applicable

**Table 2:** Comparison of the number of samples positive for intestinal protozoan parasites as detected by faecal cyst counts using microscopy (coproscopy) and by RT-PCR (*n* = 735 for all species)

Species	Positive			Negative	Positive	Positive
	COP	PCR	Both methods	Both methods	PCR/Negative COP	COP/Negative PCR
*Entamoeba histolytica*	9	12	2	716	10	7
*Giardia duodenalis*	21	105	12	621	93	9
*Blastocystis hominis*	56	479	48	248	431	8
*Dientamoeba fragilis*	na	187	na	na	na	na

*Abbreviations*: *COP* coproscopy; *na* not applicable

#### *Entamoeba histolytica*

Although the number of samples positive for *E. histolytica* using coproscopy and RT-PCR was very similar, only two samples were identified as positive by both methods, suggesting poor concordance between the methods (Table 2). However, this was not a significant difference (McNemar’s test, *χ* ^2^_1_ = 0.24, *P* = 0.63). The mean faecal cyst count recorded for the two samples that were positive by both methods was 499.5 ± 366.5 cysts/g faeces, and this was almost twice as high as the mean value of the seven samples that PCR failed to detect (267.3 ± 85.5 cysts/g faeces), four of which exceeded 200 cysts/g of faeces and one was as high as 686 cyst/g of faeces.

#### *Giardia duodenalis*

For this species, while coproscopy detected 21 positives, RT-PCR detected 105, and therefore not surprisingly there was little likelihood of detecting positives in the same subjects by each method (McNemar’s test, *χ* ^2^_1_ = 67.5, *P* < 0.001). Coproscopy detected nine positives that were not identified by RT-PCR, and for two of these high cyst counts had been recorded (3873 and 4533 cysts/g of faeces). However, there was little difference between the mean cyst count of the 12 subjects detected by both methods (1332.5 ± 211.5 cysts/g faeces) and the nine that were positive by coproscopy but not detected by PCR (1267.9 ± 568.0 cysts/g faeces).

#### *Blastocystis hominis*

For *B. hominis* there was an even greater disparity between positives detected by coproscopy and by RT-PCR (McNemar’s test, *χ* ^2^_1_ = 405.7, *P* < 0.001). Both methods agreed on just 48 positive samples, but RT-PCR detected an additional 431 positives. For this species, the mean cyst count of the samples that were positive by coproscopy but not detected by RT-PCR (635.5 ± 292.7 cysts/g faeces) was higher than that of the 48 samples deemed positive by both methods (433.9 ± 69.4 cysts/g faeces).

### The importance of inhibition in generating false negatives by RT-PCR

As shown in Table 2, some positive samples were detected only by coproscopy and not by RT-PCR. For *E. histolytica*, from the seven samples in Table 2, only five were available for reassessment *via* a modified RT-PCR method that reduced the possibility of faecal inhibition of the PCR outcome. PCR remained negative for the five samples even after removal of inhibition by including BSA in the test. For *G. duodenalis*, from the nine samples that were originally found to be positive by coproscopy but negative by PCR (Table 2), only six were still available for re-assessment. When RT-PCR was re-run, and inhibition was neutralized by the addition of BSA, amplification was observed in all six. For *B. hominis*, only four of the original eight samples were available for re-assessment and, with faecal inhibition neutralized, three gave a positive signal in RT-PCR and amplification failed in only one sample.

### Factors affecting the prevalence of infection based on detection by RT-PCR

Prevalence values based on RT- PCR at each level within the fitted explanatory factors are shown in Table 3.

**Table 3:** The effect of sex, age, region of origin and year of sampling on the prevalence % (CL_95_) of infection based on RT-PCR

	*n*	*Entamoeba histolytica*	*Giardia duodenalis*	*Dientamoeba fragilis*	*Blastocystis hominis*
Age class					
1 (18–22 years)	88	1.1 (0.08–8.40)	22.7 (13.42–35.54)	23.9 (14.24–36.69)	67.0 (54.03–78.26)
2 (23–29 years)	266	1.9 (0.91–3.82)	15.0 (11.79–18.93)	27.1 (22.86–31.73)	67.3 (62.42–71.08)
3 (30–37 years)	227	2.2 (1.18–4.04)	10.1 (7.58–13.29)	23.8 (20.02–27.97)	64.3 (59.77–68.64)
4 (38–58 years)	154	0.6 (0.07–3.97)	14.3 (9.18–21.36)	26.0 (19.03–34.05)	61.7 (53.08–69.58)
Host sex					
Males	363	0.8 (0.23–2.76)	18.2 (14.08–23.12)	24.2 (19.58–29.57)	65.6 (59.81–70.89)
Females	372	2.4 (1.13–5.03)	10.5 (7.30–14.67)	26.6 (21.70–32.15)	64.8 (58.95–70.25)
Region of origin					
West Asia	423	1.7 (0.60–4.26)	15.4 (11.29–20.48)	27.4 (22.13–33.47)	63.8 (57.53–69.74)
East Asia	223	1.8 (0.91–3.50)	9.9 (7.37–12.97)	24.7 (20.91–28.83)	58.7 (54.16–63.23)
North Africa	42	2.4 (0.17–14.00)	16.7 (7.35–32.66)	26.2 (13.95–42.72)	83.3 (67.34–92.65)
Sub-Saharan Africa	47	0 (0.00–10.60)	23.4 (11.52–40.98)	10.6 (3.35–26.30)	91.5 (76.59–97.77)
Year					
2012	196	1.5 (0.23–6.28)	26.0 (18.17–35.35)	27.6 (19.53–36.94)	69.4 (59.56–77.88)
2013	208	1.9 (1.01–3.60)	10.6 (8.06–13.66)	28.4 (24.48–32.55)	70.7 (66.42–74.61)
2014	331	1.5 (0.61–3.62)	9.7 (6.81–13.48)	22.4 (18.04–27.29)	59.2 (53.69–64.59)

#### *Entamoeba histolytica*

None of the factors significantly affected the prevalence of *E. histolytica* as assessed by RT-PCR, although the closest to significance was that for host sex (SEX × INFECTION, *χ* ^2^_1_ = 3.04, *P* = 0.081), the value for prevalence being three times higher among female subjects.

#### *Giardia duodenalis*

Prevalence of this species was significantly higher in male subjects (Table 3; SEX × INFECTION, *χ* ^2^_1_ = 16.9, *P* < 0.001) and declined significantly over time (Table 3; YEAR × INFECTION, *χ* ^2^_2_ = 35.3, *P* < 0.001), falling by 37.2% between 2012 and 2014.

#### *Blastocystis hominis*

**Fig. 1:**
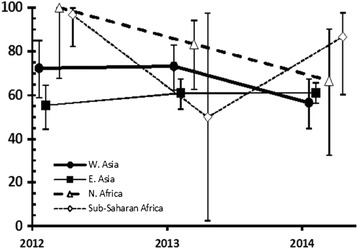
Prevalence of *Blastocystis hominis* in subjects from four regional areas, in each of the three years of the study. The wide confidence limits for subjects from sub-Saharan Africa in 2013 are attributable to the very small sample size from this region in that year (*n* = 2). The data points for subjects from different regions are offset slightly so as not to obscure the error bars

Prevalence of *B. hominis* was very similar across all four age classes and among both sexes (Table 3). However, prevalence varied significantly with region of origin of subjects (REGION × INFECTION, *χ* ^2^_3_ = 29.1, *P* < 0.001), the highest value recorded being among subjects from Sub-Saharan Africa and, with one exception, the lowest values among the two Asian groups in each year of the study (Table 3). The exception was 2013, when sample size for the Sub-Sharan Africa region was very low (*n* = 2). Prevalence also varied significantly with year of sampling (YEAR × INFECTION, *χ* ^2^_2_ = 9.5, *P* = 0.009), with the highest value recorded in 2013 and the lowest in 2014 (Table 3), but there was a weak albeit significant interaction between these two explanatory factors (REGION × YEAR × INFECTION, *χ* ^2^_6_ = 13.3, *P* = 0.039). The data illustrated in Fig. 1 show that prevalence values remained relatively steady across the 3 years among subjects from both West and East Asia with generally lower values, but changed more dynamically among those from Africa.

#### *Dientamoeba fragilis*

**Fig. 2:**
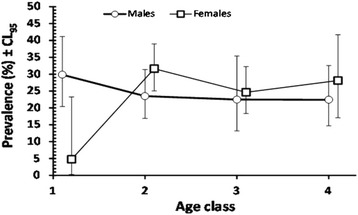
Prevalence of *Dientamoeba fragilis* in male and female subjects in each of the four age classes. The data points for the two sexes are offset slightly so as not to obscure the error bars

There was a significant effect of region of origin on prevalence of *D. fragilis* (REGION × INFECTION, *χ* ^2^_3_ = 8.52, *P* = 0.036), and this was mainly attributable to the relatively low value among subjects from Sub-Saharan Africa in contrast to the very similar values among the other three regional groups (Table 3). Prevalence varied very little among the four age classes and between the sexes (Table 3), however there was a significant interaction between the sexes and age classes (Fig. 2; SEX × AGE × INFECTION, *χ* ^2^_3_ = 9.74, *P* = 0.021). This arose because prevalence was very low among females in the youngest age class, but marginally higher prevalence in females was observed in all the older age classes.

### Comparison of infection prevalence between first-time applicant and renewal applicant

In a model that also took account of the significant effect of host sex and of year of sampling (see above), the prevalence of infection with *G. duodenalis* was higher among first-time applicants compared with renewal applicants (Table 4), but the difference was not statistically significant (IMMIGRATION STATUS × INFECTON, *χ* ^2^_1_ = 3.1, *P* = 0.079). When the 47 subjects applying for renewal were scrutinised further, prevalence was actually higher, although not significantly so (*P* = 0.44), among those applying for renewal for the first time (*n* = 13; 15.4% [CL_95_ = 2.81–43.39]) compared with 14.5% (Table 4) among first-time applicants. Our data contained only 34 subjects who applied for renewal of their permits for more than the first time (2nd to 12th time) and among these prevalence was lower still (8.8% [CL_95_ = 3.08–20.92]).

**Table 4:** Prevalence % (CL_95_) of infections based on RT-PCR and immigration status of subjects

Species	1st time applicants^a^(*n* = 688)	Renewal applicants^b^ (*n* = 47)
*Giardia duodenalis*	14.5 (12.15–17.25)	10.6 (3.35–26.30)
*Blastocystis hominis*	64.8 (61.31–68.19)	70.2 (52.56–83.51)
*Dientamoeba fragilis*	26.3 (23.24–29.61)	12.8 (4.59–28.56)

^a^Persons arriving for the first time in Qatar

^b^Persons renewing work permits

For *B. hominis*, prevalence was marginally higher among renewal-applicants (Table 4) and again not significantly different from the first-time applicants (in a model that included YEAR and REGION, for IMMIGRATION STATUS × INFECTON, *χ* ^2^_1_ = 0.68, *P* = 0.41).

However, the prevalence of *D. fragilis* differed significantly between first-time and renewal-applicants (IMMIGRATION STATUS × INFECTION, *χ* ^2^_1_ = 4.87, *P* = 0.027), and the data in Table 4 show that prevalence was just less than half among the latter, compared with the former group of subjects. However, in a model that also included host sex, age and region of origin of the subjects, two significant interactions were detected. The first was with host sex (SEX × IMMIGRATION STATUS × INFECTION, *χ* ^2^_1_ = 6.6, *P* = 0.010). The difference in prevalence between female first-time and renewal applicants (Table 5; 32.3%) was less than that among male subjects (56.7%). To explore the effect of region of origin of subjects, analysis was also conducted on a smaller database that excluded the African subjects because there were no applicants for renewal among the 42 North Africans and only one among the Sub-Saharan Africans. This showed that there was a significant interaction with region of origin of the subjects (REGION × IMMIGRATION STATUS × INFECTION, *χ* ^2^_1_ = 9.6, *P* = 0.002). Prevalence of *D. fragilis* was 37.6% lower among West Asian renewal applicants compared with first-time applicants (Table 5), but among East Asians this was 100%, although the sample size was small.

**Table 5:** Prevalence % (CL_95_) of *Dientamoeba fragilis* among male and female, Western Asian and Eastern Asian, subjects

	1st time applicants		Renewal applicants	
	*n*	Prevalence	*n*	Prevalence
Males	327	25.7 (21.15–30.76)	36	11.1 (4.37–24.28)
Females	361	26.9 (22.02–32.35)	11	18.2 (3.34–50.00)
Western Asia	389	28.3 (23.14–34.04)	34	17.6 (8.79–31.56)
Eastern Asia	211	26.1 (22.28–30.23)	12	0 (0.00–24.26)

*Note*: Data for North and sub-Saharan Africans have been excluded from the regional analysis (the two lower rows in the Table 3) because there were no applicants for renewal among the 42 North Africans and only one among the sub-Saharan Africans

### The effect of job family on the prevalence of infection based on RT-PCR

First, all statistical models were based on fitting just JOB FAMILY and INFECTION (Table 6). Only the prevalence of *G. duodenalis* varied significantly between job families (see Table 6; JOB FAMILY × INFECTION, *χ* ^2^_3_ = 13.4, *P* = 0.004), so in this case a model was also fitted that included significant effects from the first round of analyses above and this included SEX, YEAR, JOB FAMILY and INFECTION. The only term that included JOB FAMILY and INFECTION, also included YEAR, so there was also significant variation between years (YEAR × JOB FAMILY × INFECTION, χ^2^_6_ = 13.6, *P* = 0.035). Examining this more closely in 2012, 2013 and 2014, prevalence was relatively steady across this period among pink/white collar workers (11.5%, 9.1% and 13.8%, respectively), fell moderately with time among house maids (16.7%, 8.5% and 5.8%, respectively), and more sharply among both blue collar workers (52.0%, 11.7% and 13.7%, respectively) and among the food workers (33.3%, 21.4% and 0%, respectively).

**Table 6:** The effect of job family on the prevalence % (CL_95_) of infection based on RT-PCR

	*n*	*Giardia duodenalis*	*Dientamoeba fragilis*	*Blastocystis hominis*
Blue collar workers	273	20.1 (16.39–24.52)	27.1 (22.85–31.83)	64.8 (59.89–69.52)
Pink & white collar workers	66	12.1 (6.45–21.29)	15.2 (8.45–24.97)	66.7 (55.41–76.41)
House maids	353	9.9 (6.92–13.88)	26.9 (22.12–32.33)	64.6 (58.92–69.91)
Food workers	43	16.3 (6.99–32.40)	18.6 (8.73–35.00)	69.8 (52.98–82.69)
Statistical analysis				
*χ* ^2^_3_		13.4	6.04	0.54
*P*		0.004	0.11 (ns)	0.91 (ns)

*Abbreviation*: *ns* not significant

## Discussion

In the current paper we assessed the RT-PCR method for detecting intestinal protozoan infection and compared its efficiency with traditional coproscopy. We then used prevalence data derived by the molecular approach to identify host intrinsic and extrinsic factors that may have influenced prevalence of each of the species in our study.

Comparing prevalence values based on coproscopic analysis with those derived by our molecular tools, a huge net difference is apparent in our data-sets. Based on RT-PCR, we have previously already reported an enormous difference in the efficiency of detecting *B. hominis* infections; 71.1% prevalence based on RT-PCR compared to only 6.9% by conventional coproscopy [10]. The prevalence values obtained in the current study after analyzing different subjects are in close agreement, with 65.2% *vs* 7.6% for the molecular and microscopy tools, respectively. Similar results in detecting intestinal protozoa have been reported elsewhere in Brazil, Australia and Denmark [17–19], although the difference between the two methodologies was not as disparate as our experience [20].

Despite the wide-scale and continuing use of traditional coproscopy for examination of stool samples in epidemiological surveys, it is now clearly apparent that this method vastly underestimates the actual prevalence of protozoan infections in communities. Coproscopy is considered nowadays to be less sensitive and less specific than detection by PCR. The utility of continuing to employ conventional coproscopy in this field is therefore highly questionable, and there is an urgent need for the wider implementation of alternative methods based on molecular tools. These already have proven efficiency and reliability, but are being refined further by researchers introducing new procedures and techniques to improve and enhance sensitivity and performance.

Indeed, although methods based on PCR have replaced traditional pathogen detection assays in many clinical microbiology laboratories, and despite their acknowledged superior sensitivity, these molecular tools are not foolproof and have some weaknesses that still limit their use in diagnostics. The most important of the problems encountered with clinical samples is the occurrence of false negative results [21]. Some samples revealed by routine coproscopy to be positive, i.e. they contain clear evidence of the presence of transmission stage of the parasites in question, can still fail to be detected by PCR, as observed in our current study. In the case of *E. histolytica* seven samples recorded as positive by coproscopy were negative by RT-PCR, and these were all double checked, with relevant controls, for a signal in RT-PCR but with lack of success. One possibility may be the specificity of the molecular tools used for detection for *E. histolytica* which would not have detected other *Amoeba* species (e.g. *E. dispar*, *E. moshkovskii* and *E. bangladeshi*) whose transmission stages are morphologically identical when viewed by coproscopy [22]. In general, there is a consensus among researchers that the occurrence of false-negative cases may be associated also with the absence or low concentration of DNA in samples submitted for extraction and amplification of genetic material, although this is unlikely to be the case when cyst concentration is high, as observed in some of our samples. False negatives, especially in the diagnosis of pathogenic agents, have significant consequences for both treatment and rapid containment of disease in affected subjects and in populations. They may be due to the presence of inhibitors in faeces resulting in non-amplification of the targeted gene fragments [17], and this appears to be perhaps the most important complication, limiting the efficiency of PCR-based techniques. It is suggested that the frequency of occurrence of PCR inhibitors in human faeces is higher in the adult populations compared to that in pediatric populations [23]. The concentration of these inhibitors in stool samples is correlated with differences in gut microbiota, dietary or other factors in our environment or stemming from our modern lifestyles [21].

A variety of different inhibitors are known to negatively impact PCR sensitivity and hence impede pathogen detection [24]. Interference with cell lysis, sequestration or degradation of nucleic acids and hindrance of polymerase activity are all common mechanisms of PCR inhibition. Complex polysaccharides, bilirubin and bile salts found in stools can block the polymerase’s active site [25]. Several methods for the removal of inhibitors or for the reduction of their effects have been proposed. Selection of an appropriate method for sample processing and nucleic acid extraction is one obvious starting point. It may include the choice of a more robust DNA polymerase and/or use of specific PCR additives. DNA extraction by kits using “spin columns” can reduce the concentration of PCR inhibitors, as well as enabling greater efficiency in obtaining the DNA sample. However, some inhibitors can be co-eluted with the DNA [17]. A more general and widely applied method is the dilution of the sample or the extracted nucleic acid, which will automatically result in a dilution of the PCR inhibitors. However, such dilution is likely also to be accompanied by a reduction in sensitivity [24]. Another approach is to add to the PCR mixture substances that will reduce PCR inhibition as for example: BSA, betaine, formamide, glycerol, gp32, nonidet P40 and tween. Other methods that neutralize inhibitors focus on modification of DNA polymerase such as deletion of the N-terminal portion of the DNA polymerase [25]. In our current analysis, BSA was efficient in removing the inhibition in almost all of the investigated cases of false negatives.

Moreover, besides these useful measures, PCR inhibition should be monitored by a suitable internal amplification control (IAC) to avoid false-negative results, especially in clinical laboratories. IAC is a non-target nucleic acid sequence that is simultaneously co-amplified with the primary target sequence in the same PCR tube. Internal controls could be designed to monitor the sample preparatory step/s alone or the PCR amplification step alone, or both [26].

Notwithstanding the false negatives in the present study, we utilized our Medical Commission data-base containing records of the same stool samples and relevant information about the subjects themselves. We re-examined some of the intrinsic and extrinsic factors that may have affected prevalence of these protozoan infections in the population, this time basing presence of infection solely on positivity in our RT-PCR assay. Available information for each individual included whether he/she was a recent arrival or a resident foreign immigrant worker, and in this context our study has provided results that are consistent with earlier studies that were based solely on coproscopic analyses [27].

*Blastocystis hominis* was the most frequently identified protozoan with a prevalence based on RT-PCR that was close to previously reported values [10]. Similar high prevalence was observed using the RT-PCR method (88.7%) among an indigenous population from the Colombian Amazon Basin [28]. The distribution of *B. hominis* infections among investigated populations suggests spatial heterogeneity with respect to host age and sex. Such inconsistencies are most likely attributable to local factors such as environmental conditions that influence the extent of, and the efficiency of the faecal-oral route of transmission among host sectors of varying age, and between the two sexes [29]. The most prominent source of variation in prevalence of *B. hominis* that we detected here was in the region of origin of the subjects, with immigrants from Africa showing the highest prevalence and those from Eastern Asia the lowest, supporting earlier findings [10]. The prevalence of *B. hominis* infection is known to vary from country to country, as well as within countries [30], with the highest prevalence ever reported worldwide (100%) for this parasite observed in a Senegalese population [31]. Prevalence was virtually identical in both long-term residents, applying for renewal of their work permits and among first-time applicants and there was no convincing directional change over time such as that observed previously over a much longer time frame [32], supporting the occurrence of local transmission of this species.

The second most prevalent protozoan identified was *D. fragilis,* a species that we have not analyzed previously because its transmission stages are difficult to detect by microscopy. Detection by molecular tools provided the first prevalence data for this species, as coproscopic identification is not reliable. Indeed, it depends mainly on the triple faeces test (TFT), which entails collection of subject’s stools on three consecutive days. It is crucial that a sample for diagnosis is taken on at least one of the 3 days, at a time when there are intestinal symptoms, and the stool is abnormal. As its name indicates, *D. fragilis* is extremely fragile. The parasite dies outside the host body almost immediately, and is rapidly broken down by bacteria and enzymes and decomposes, making coproscopic identification extremely difficult and in most cases unlikely. The prevalence of *D. fragilis* has been found to vary widely between surveys, ranging from 0.3–89% depending on age and the methods adopted for diagnosis [33]. Similar prevalence to ours was reported in Italy (21.4%) among patients suspected of having an intestinal parasitosis [34]. We found that while there was no difference in prevalence between the sexes or between age classes, there was a significant interaction arising from the relatively low prevalence among very young female subjects and the contrasting high prevalence among male subject in this age class comprising subjects aged 16–22 years. Immigrant female workers generally take up jobs such as house maids or in the catering/food-handling industries. Regular annual renewal of work permits is mandatory for the food-handlers which may explain the lower prevalence of protozoan infections, suggesting greater attention given to the anti-parasite therapy and prophylaxis [27].

In contrast to the other species *G. duodenalis* showed a marked and significant fall in prevalence across the three years of the study, and unlike in our earlier study based on data collected over a decade, here there was no significant decline in prevalence with host age. Nevertheless, it is worth pointing out that despite our sample size being much smaller here than previously, prevalence values were numerically higher in the youngest age class, and that analysis of the age effect was only just the wrong side of significance (*χ* ^2^_3_ = 7.03, *P* = 0.071). Persistence of *G. duodenalis* infection among young male subjects in Qatar has been suggested to indicate continued exposure and the affected individuals are most likely to be the unskilled construction workers aggregating in overcrowded labour camps, where conditions may be cramped and sanitary facilities stretched [32]. Our current data showed that blue collar workers had the highest prevalence among the five job families. Local transmission in Qatar of *G. duodenalis* is of concern especially among the food-handlers, a group at particular risk of disseminating infections further and among whom prevalence was also relatively high compared to house maids and pink and white collar workers.

## Conclusions

There is therefore a need for better awareness about these parasites and about strategies for limiting their infection both in Qatar and in the countries of origin of the immigrant population. Clearly the RT-PCR method for detecting intestinal protozoan infections is far more efficient than coproscopy, although, as we have indicated, there is further room for improvement of efficiency by eliminating the false negatives in PCR assays. Nevertheless, despite this limitation of the RT-PCR, it is far more efficient at detecting positives in all the species that were examined in the current work, with the added advantage of detection of *D. fragilis*, which cannot be detected usually by coproscopic examination. Therefore, based on our findings, we strongly recommend that henceforth intestinal protozoa should be screened by RT-PCR with the use of BSA, rather than the traditional coproscopic examination. Immigrant food-handlers should remain a particular focus of the health authorities. Frequent health checks and screening of stool samples should be consolidated as a condition for the renewal of their work permits, as they present a particularly high risk of disseminating infections locally in the population of Qatar.
